# SerThr-PhosphoProteome of Brain from Aged PINK1-KO+A53T-SNCA Mice Reveals pT1928-MAP1B and pS3781-ANK2 Deficits, as Hub between Autophagy and Synapse Changes

**DOI:** 10.3390/ijms20133284

**Published:** 2019-07-04

**Authors:** Georg Auburger, Suzana Gispert, Sylvia Torres-Odio, Marina Jendrach, Nadine Brehm, Júlia Canet-Pons, Jana Key, Nesli-Ece Sen

**Affiliations:** Experimental Neurology, Goethe University Medical Faculty, 60590 Frankfurt am Main, Germany; gispert-sanchez@em.uni-frankfurt.de (S.G.); torresodio@medicine.tamhsc.edu (S.T.-O.); marina.jendrach@charite.de (M.J.); nadine.brehm85@googlemail.com (N.B.); jcanetpons@gmail.com (J.C.-P.); key@stud.uni-frankfurt.de (J.K.); nesliecesen@gmail.com (N.-E.S.)

**Keywords:** Parkinson’s disease, brain phosphorylome, PINK1, alpha-synuclein, microtubular cytoskeleton, autophagy, synaptic signaling

## Abstract

Hereditary Parkinson’s disease (PD) can be triggered by an autosomal dominant overdose of alpha-Synuclein (SNCA) as stressor or the autosomal recessive deficiency of PINK1 Serine/Threonine-phosphorylation activity as stress-response. We demonstrated the combination of PINK1-knockout with overexpression of SNCA^A53T^ in double mutant (DM) mice to exacerbate locomotor deficits and to reduce lifespan. To survey posttranslational modifications of proteins underlying the pathology, brain hemispheres of old DM mice underwent quantitative label-free global proteomic mass spectrometry, focused on Ser/Thr-phosphorylations. As an exceptionally strong effect, we detected >300-fold reductions of phosphoThr1928 in MAP1B, a microtubule-associated protein, and a similar reduction of phosphoSer3781 in ANK2, an interactor of microtubules. MAP1B depletion is known to trigger perturbations of microtubular mitochondria trafficking, neurite extension, and synaptic function, so it was noteworthy that relevantly decreased phosphorylation was also detected for other microtubule and microfilament factors, namely MAP2^S1801^, MARK1^S394^, MAP1A^T1794^, KIF1A^S1537^, 4.1N^S541^, 4.1G^S86^, and ADD2^S528^. While the MAP1B heavy chain supports regeneration and growth cones, its light chain assists DAPK1-mediated autophagy. Interestingly, relevant phosphorylation decreases of DAPK2^S299^, VPS13D^S2429^, and VPS13C^S2480^ in the DM brain affected regulators of autophagy, which are implicated in PD. Overall, significant downregulations were enriched for PFAM C2 domains, other kinases, and synaptic transmission factors upon automated bioinformatics, while upregulations were not enriched for selective motifs or pathways. Validation experiments confirmed the change of LC3 processing as reflection of excessive autophagy in DM brain, and dependence of ANK2/MAP1B expression on PINK1 levels. Our new data provide independent confirmation in a mouse model with combined PARK1/PARK4/PARK6 pathology that MAP1B/ANK2 phosphorylation events are implicated in Parkinsonian neurodegeneration. These findings expand on previous observations in *Drosophila melanogaster* that the MAP1B ortholog futsch in the presynapse is a primary target of the PARK8 protein LRRK2, and on a report that MAP1B is a component of the pathological Lewy body aggregates in PD patient brains. Similarly, *ANK2* gene locus variants are associated with the risk of PD, ANK2 interacts with PINK1/Parkin-target proteins such as MIRO1 or ATP1A2, and ANK2-derived peptides are potent inhibitors of autophagy.

## 1. Introduction

Parkinson’s disease (PD) is the second most frequent neurodegenerative disorder. Although its most important risk factor is old age, there are also genetic variants that exacerbate the risk [1].

In patients without familial inheritance, genome-wide surveys have implicated dozens of genes in the pathogenesis, with the biggest impact being due to single nucleotide polymorphisms within the alpha-synuclein gene (gene symbol *SNCA*). Thus, any sporadic PD patient with typical manifestation after the age of 70 years also carries a certain mutation burden. 

Some 5%–10% of patients report a positive family history. Among them, some monogenic traits were identified in rare families across the world. Hereditary variants of PD trigger early manifestation and often rapid progression. The A53T mutation in alpha-synuclein was first identified and is responsible for the PARK1 variant of PD, with onset age around 50 years and autosomal dominant inheritance. Gene triplication and duplication of the alpha-synuclein gene without missense-mutation also causes PD via gene dosage effects, with clinical onset after 30 years and 50 years, respectively. In contrast, a reduced alpha-synuclein dosage protects against PD [2]. Autosomal dominant pedigrees also led to the identification of the *LRRK2* gene as the most frequent cause of genetic PD (PARK8), but the manifestation age is usually later and the penetrance is limited, so it is harder to explore its mutation effects in disease models.

Very early onset is observed in juvenile PD with autosomal recessive inheritance. Mutations in the Parkin gene are the most frequent cause identified in cases manifesting around age 25 (PARK2 variant of PD), while mutations in PINK1 are less frequent (PARK6 variant) [3,4]. Despite their early onset, such patients show a mild phenotype and progression with sustained good responses to dopaminergic treatment, unless additional stress is present. This severity difference between recessive and dominant PD is exemplified by the good sleep quality in PARK6, versus the severe REM sleep behavior disorder in PARK1/PARK4 cases already at presymptomatic stages [5,6].

PINK1 and Parkin together coordinate mitochondrial quality control after age- or stress-induced damage. Mitochondrial dysfunction leads to the kinase PINK1 becoming abundant at the mitochondrial outer membrane, which starts to phosphorylate ubiquitin and attracts the ubiquitin E3 ligase Parkin from the cytosol [7]. A loss-of-function of PINK1 can be rescued by Parkin [8]. Together, PINK1 and Parkin target the GTPase MIRO to trigger the microtubular removal and autophagic degradation of the damaged mitochondrial segment [9]. 

Thus, post-translational modifications like the phosphorylation activity of PINK1 and the ubiquitylation activity of Parkin are governing the mitochondrial autophagy pathway [10], which is at the heart of typical PD with early-onset autosomal recessive inheritance. Advances in global proteome techniques by mass spectrometry have already made it possible to document the Parkin-dependent ubiquitination events, initially only in tumorous peripheral cell lines [11]. Although intense efforts were made to define the molecular targets of PINK1 and Parkin also in neuronally reprogrammed cells and neural cell lines, so far no neuron-specific phosphorylation and ubiquitination events could be demonstrated by this in vitro approach. Recently we documented the global ubiquitylome of aged brain in Parkin-deleted mice and could thus demonstrate how the altered turnover of neuron-specific factors and disturbed calcium homeostasis combine to impact neural firing frequency [12].

Here, we attempted to define the global phosphorylome in the aged brain from a mouse model of PD. The goal of the study was the identification of such additional PINK1-controlled proteins, which may be exclusively present in differentiated and aged brain neurons. These efforts are important, given that PINK1 is not only controlling mitophagy, but also coordinates the resynthesis of damaged mitochondrial proteins [13], the repair of mitochondria via fusion–fission dynamics [14], mitochondrial apoptosis [15], and also, to some degree, bulk autophagy beyond mitochondria [16], and particularly neuroinflammation [17]. For this phosphorylome survey, we used PINK1-deficient animals that we had generated and characterized, demonstrating that they have mitochondrial dysfunction but no cell loss in their brains within their lifespan [18]. This dysfunction leads to a subtle phenotype involving altered neuronal excitability and calcium homeostasis in corticostriatal projections leading to hypersynchrony, as well as a dysfunction of dopamine release in the midbrain neurons [19,20,21]. Given that the PINK1 protein is only stable during cell stress periods and that toxins like CCCP or starvation are needed, so as to maximize PINK1 abundance and subsequent phosphorylation events [22,23,24,25], we bred a mitochondrial stress factor into the PINK1-deficient mice. 

Regarding the role of different stressors, the mitochondrial damage is particularly strong in the flight muscles of *D. melanogaster* flies with ubiquitous PINK1-deletion, due to the continuous energetic stress [26,27]. A recent mouse study demonstrated that PINK1-/Parkin-dependent neuroinflammation and neurodegeneration can be rescued by deletion of the gene *STING*, employing PINK1-/Parkin-knockout animals that were stressed either by exhaustive exercise or by crossbreeding with mutator mice with accumulation of mtDNA damage [28]. We favored the crossbreeding with mice that overexpress A53T-alpha-synuclein as stress condition, for several reasons. Firstly, alpha-synuclein is a Parkinson-specific stressor [29]. Secondly, alpha-synuclein has a physiological localization at the interface between mitochondria and endoplasmic reticulum colocalizing with MIRO1, and exerts a powerful mitochondrial stress upon its overexpression [30,31,32]. Thirdly, PINK1-deficient patient skin fibroblasts show transcriptional induction of alpha-synuclein, and PINK1-deficient mice showed enhanced alpha-synuclein immunoreactivity in the brainstem [18,33,34,35], so there seems to be a genetic interaction between both factors. 

The A53T-SNCA overexpressing mice employed are a useful PD model, given that they showed (1) no loss of neuronal cell bodies within their lifespan, but did show (2) a progressive deficit of spontaneous locomotion, (3) impaired dopamine signaling, increased postsynaptic sensitivity to dopamine-agonists, with altered synaptic plasticity in the striatum, (4) diminished synaptic vesicle release with expression dysregulation of synaptic vesicle dynamics factors, (5) elevated intrinsic pacemaker frequency in dopaminergic midbrain neurons, (6) and altered levels of several alpha-synuclein-homologous 14-3-3 isoforms that modulate phosphorylated proteins [36,37,38,39,40,41,42,43,44,45]. 

Our crossbreeding of PINK1-deficient mice with A53T-SNCA mice reduced survival and potentiated their locomotor deficit, triggering the altered ubiquitination of alpha-synuclein and the increased formation of pSer129-alpha-synuclein aggregates [46]. Beyond the ubiquitylome study in these double-mutant (DM) mice, we assessed other post-translational modifications in the global proteome from aged brains, demonstrating (1) that several mitochondrial respiration factors showed an 8-fold reduction of lysine acetylation, and (2) that mitochondrial biogenesis factors showed 1.5–4-fold inductions of arginine methylation [47,48]. Here, we also quantified Ser/Thr-phosphorylation in the global proteome from aged brain in these DM mice.

## 2. Results and Discussion

**Figure 1:**
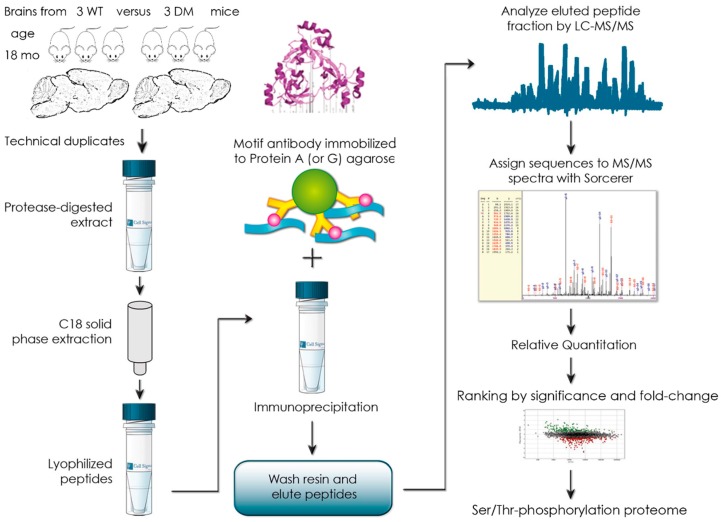
Flow chart illustrating the experimental approach to measure the Ser/Thr-phosphorylation of trypsination-derived peptides from the global proteome of mouse brain. C18 solid phase extraction, lyophilization, immunoprecipitation with motif antibody (3D structure illustrated) immobilized on protein A (or G) agarose (beads shown as green circle, immunoglobulin coating in yellow, binding of digested peptides in blue with their Ser/Thr-phosphorylations in pink), and mass spectrometry were used. Graphic elements from internet sites were used with permission of Cell Signaling Inc., see http://www.cellsignal.com/common/content/content.jsp?id=proteomics-discovery.

To document the abundance of Ser/Thr-phosphorylation motifs (PhosphoScan^®^) in the global brain proteome, a quantitative label-free mass spectrometry approach was used. In view of the importance of ageing as a risk factor for PD, 18-month-old DM mice were chosen, comparing three of them versus three age-/sex-matched wildtype (WT) mice (see Figure 1). Raw data were processed by filtering for consistency and effect size: To avoid inconsistencies, factors were excluded if each of the three DM mice did not show the same direction of change. Furthermore, factors were excluded if a two-fold change was not detected at least once among the biological triplicates. The remaining observations comprised 45 factors with relevant phosphorylation downregulations at one or several residues, and 49 factors with relevant upregulations (see Table S1).

### 2.1. Strongest Downregulations Affect Microtubular Functions

As shown in Table 1, a massive (>−300-fold) reduction was observed for the uncharacterized phosphorylation-site pT1928 within MAP1B (microtubule-associated protein 1B). This observation reflected a highly specific effect, since many other established phospho-residues in this intensely phosphorylated protein did not exhibit relevant changes: Within MAP1B, eight Ser/Thr-sites are known to be controlled by CDK5, seven by GSK3β, six by p38MAPK, five by CKII, two by CDC2, two by PKA, one each by ERK1 and PKG, while its light-chain LC1 and its heavy-chain region containing an 17mer imperfect repeat seem not targeted by these kinases [49]. Known phosphorylations of MAP1B, particularly at the GSK3β-dependent sites pS1260/pT1265, are mostly located in the interaction domains, where they modulate the stability of microtubules together with the mobility of microfilaments and growth cones [49]. 

The second biggest reduction (average −3.3-fold) was observed for ANK2 isoforms 2 and 3 (residue pS3781) (Table 1). It appeared even more massive in the analysis of the DM2 mouse (−715-fold) but showed less consistency than MAP1B among the biological triplicates. This phospho-site was previously reported to change during long-term potentiation and in fronto-temporal lobar degeneration [50,51]. Again, other established phosphorylation sites in ANK2 that are under control e.g., of CK2 [52] were unchanged. The phosphorylation losses in specific residues of MAP1B and ANK2 seem not to be random effects, since both factors interact physically and exert joint functions. 

The association between ANK2 isoforms and MAP1B together with spectrin in *D. melanogaster* was reported to form a membrane-associated microtubule-organizing complex that determines axonal diameter, supports axonal transport, and provides independent control of synaptic dimensions and stability [53,54]. Ankyrins also stabilize the membrane position of Na^+^/K^+^ pumps and in particular ANK2 controls ATP1A2 [55,56]. Together, ankyrins with adducin isoforms and the 4.1 protein family link via spectrin onto the actin cytoskeleton, in order to determine the cell surface presence of membrane receptors and channels [57,58]. 

Thus, it is relevant that ADD2 (beta-adducin) showed a 1.7-fold increase at pS528/pS530/pS532/pT533/pS535 in DM brains (Table 1). This putative pathway dysfunction (illustrated in Figure 3A) is also supported by the −2.1-fold decrease of pS86 in 4.1G protein (EPB4.1L2) together with 2.2-fold increases of pS541, pS544, and pS546 in 4.1N protein (EPB4.1L1) that were documented in DM brains. The protein 4.1G regulates the membrane presence of receptors (e.g., mGluR1a, A1 adenosine receptor, Ca^2+^-ATPase SERCA2, Fc gamma RI) and of cell adhesion molecules (CADM4, PTA-1) (Figure 3A). The protein 4.1N regulates the membrane accumulation of AMPA glutamate receptors GluR1 and GluR4, the dopamine D2 and D3 receptors, the inositol 1,4,5-trisphosphate receptor (ITPR1), as well as the cell adhesion molecules CADM3 and CADM1 [57]. 

Within the neurite cytosol on the other hand, ANK2 and the established PINK1-/Parkin-target MIRO1 [9] are both required to stabilize APC2 at microtubule branch points and to control the axonal transport of mitochondria to such points within neuronal dendrites [59,60,61]. Similarly, MAP1B modulates the axonal transport of mitochondria [62], and its light chain LC1 turnover is controlled by the mitophagy regulator MARCH5 [63]. 

How are these findings relevant for PD? Importantly, it was found that alpha-synuclein associates with MAP1B (previously known as MAP5) and sequestrates it into the pathological aggregates known as Lewy bodies in PD brains [64,65]. Thus, it is conceivable that the deficient phosphorylation of MAP1B in the DM brain is caused by association of its 17-mer repeat region with pathological alpha-synuclein and consequent masking of the pT1928 site, rather than the loss of PINK1 phosphorylation activity. The kinase LRRK2 is responsible for the autosomal dominant PARK8 variant of PD and was shown in *D. melanogaster* flies to phosphorylate the MAP1B ortholog futsch at the pSer4106 site, far beyond the sequence that is conserved in mammals as C-terminus [66,67]. It is crucial to note that MAP1B-LC1 was found stably associated with LRRK2 upon yeast-two hybrid studies and that MAP1B-LC1 overexpression rescued LRRK2-mutation triggered cytotoxicity in human neuroblastoma cells [68]. It is also important to know that the latest systematic investigation of genetic risk factors among >13,000 PD patients and 95,000 control individuals in a genome-wide association study meta-analysis [29] identified the variant rs78738012 next to the *ANK2* gene with high significance (joint *p* = 4.78 × 10^−11^), providing independent evidence that ANK2 alterations contribute to the pathogenesis of Parkinson’s disease. Furthermore, the ANK2-controlled ATP1A2 was recently identified in our ubiquitylome profiling of aged brains from the PARK2-mouse model as a putative target of Parkin-dependent ubiquitylation [12].

Overall, the phosphorylome survey highlighted a cluster of dysregulations in the cytoskeletal machinery that connects the energy-producing mitochondria with the energy consuming plasma membrane signaling apparatus. The novel and neuron-specific strong phosphorylation deficits in MAP1B and ANK2 appear to represent a functional context. They also provide independent confirmatory evidence for their involvement in PD. Thus, they were pursued further.

### 2.2. Phospho-Residues of MAP1B and ANK2 Are Conserved and Fit with PINK1-Target Criteria

**Figure 2:**
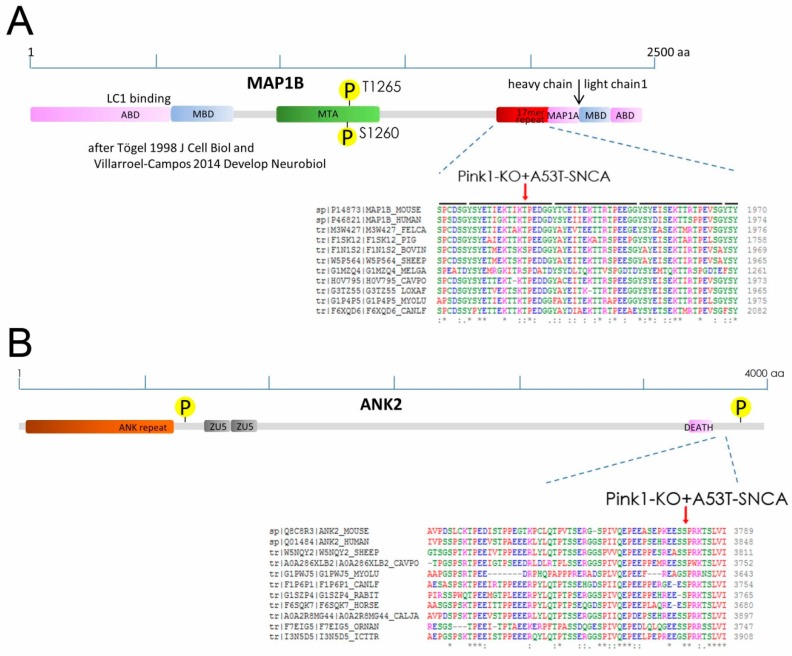
(**A**) Scheme of MAP1B protein sequence, cleavage into heavy chain and light chain 1 (LC1), structural motifs with their interactions (LC1 binding in heavy chain, actin binding domain ABD, microtubule binding domain MBD, microtubule assembly helping site MTA, region that contains 12 repeats of a 17mer sequence, MAP1A interacting domain), and characterized GSK3beta-dependent phospho-sites at pS1260/pT1265, which have an established effect on cytoskeletal reorganization. Alignment of MAP1B protein sequences across species illustrates the conservation of the DM-modulated Thr/Ser site (red arrow) within the 17mer repeat (highlighted as black bars above sequence) of unknown function. The symbols * : . below the amino acid alignment highlight different phylogenetic conservation. (**B**) Scheme of ANK2 protein sequence, structural motifs (Ankyrin repeat, ZU5 domains, and DEATH domain, with characterized phosphorylation sites at S846 and S3850. Alignment of ANK2 protein sequences across species illustrates the conservation of the DM-modulated Ser site (red arrow) within the C-terminal target interaction domain. For a complete list of the residues with potential phosphorylation, see www.phosphosite.org.

The MAP1B site pT1928 is located within the C-terminal part of the heavy chain, which contains 12 repetitions of the 17mer sequence YSYETXEXTTXXPXXXX (aa 1866–2069), whose function is presently not understood. Although there is a lot of sequence variability in this imperfect repeat before the conserved Proline, within the fourth repetition the residue 1928 before the conserved Proline has a conserved Ser/Thr phosphorylation site according to phylogenetic analyses by CLUSTAL OMEGA (see Figure 2A). 

Similarly, the ANK2 residue 3781 also has a conserved Ser phosphorylation site before a conserved Proline (see Figure 2B). Interestingly, it is known that this ANK2 C-terminal regulatory region beyond the DEATH domain is responsible for interaction with specific targets such as ITPR1 or RYR2 to be anchored in the membrane [69]. 

Importantly, previous in vitro analyses have revealed PINK1 to phosphorylate synthetic peptides exclusively at Ser/Thr residues, with strong preference for a neighboring proline at the +1 position [70]. The target sequences TIKT*PED in MAP1B and EESS*PRK in ANK2 both show a proline at the +1 position after each phospho-residue (Figure 2), so they are a good fit with these PINK1 substrate specificity criteria. Overall, these data suggest that MAP1B and ANK2 could be targets of PINK1 kinase.

### 2.3. Pathway Enrichments Highlight Microtubule Functions, Synaptic Signaling, and Kinase Domains

**Figure 3:**
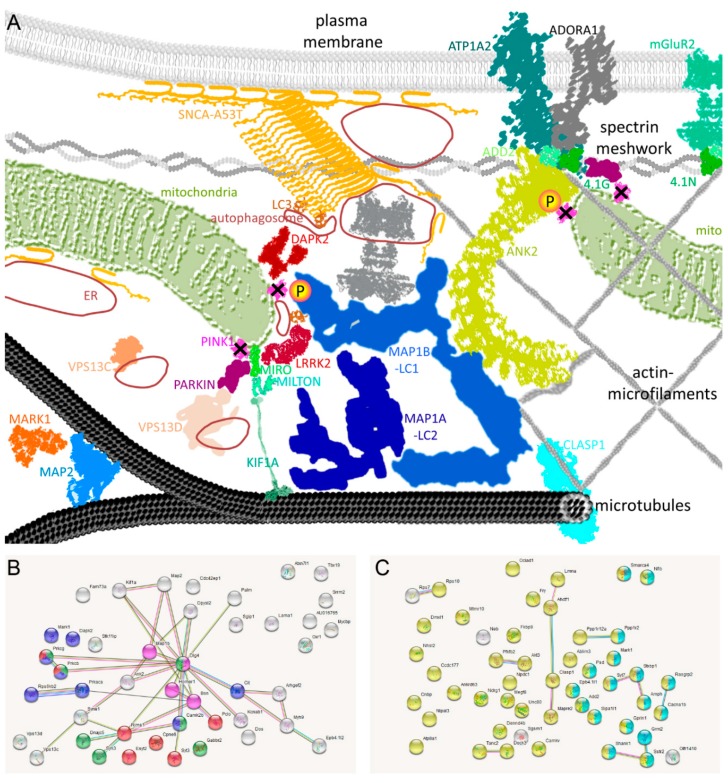
(**A**) Schematic representation of dysregulated factors, based on their subcellular localization and on textmining about functional interaction effects. Three cytoskeletal layers underlying neuronal plasma membranes (microtubules, actin-microfilaments, spectrin meshwork) are shown, which provide correct localization to mitochondria, vesicles, and the membrane channels/pumps. The overexpressed mutant A53T-SNCA with its aggregates, the deleted PINK1 kinase, and the downstream proteins affected by altered phosphorylation—and LRRK2 as a DAPK2 homologous kinase that phosphorylates MAP1B in abnormal manner in the PARK8 variant of PD—are visualized as colored symbols, reminiscent of their known 3D structures and with rough representation of their size differences. The two strongest deficits in phosphorylation are highlighted in pink/yellow circles. (**B**) As additional automated scheme on protein–protein interactions and pathway enrichments, search tool for the retrieval of interacting genes (STRING) diagrams are provided for all 45 factors with reduced Ser/Thr-phosphorylation and (**C**) 49 factors with increased Ser/Thr-phosphorylation. The protein–protein enrichment *p*-value was very significant among the downregulations (*p* = 7.54 × 10^−12^), but barely significant among the downregulations (*p* = 0.036). Previous knowledge of the interaction between these factors from experiments, co-expression, or text mining is illustrated by connecting lines of various colors.

MAP1B deficiency alone has several detrimental effects on the microtubule support for synaptic function [71]. It is therefore noteworthy that various other microtubule-related factors showed dysregulated phosphorylation sites in DM brains. The factors MAP2^S1801^, MARK1^S394^, MAP1A^T1794^, and KIF1A^S1537^ decreased up to −2.3-fold, while CLASP1^S646^ increased as a factor that tethers microtubules to the extracellular matrix, remodeling microtubules according to signals and damage [72,73] (see Table 1). The clustering of phosphorylation dysregulations among factors that constitute the neurite cytoskeleton, their interaction with mitochondria, and their anchoring of the membrane signaling machinery was evident, so we attempted to illustrate this subcellular enrichment in Figure 3A. In addition, downregulated phospho-sites were documented for 11 presynaptic (AMPH, BSN, CACNA1B, DNAJC5, CRMP-2, KCNAB1, PICCOLO, RIMS1, STXBP1, SYN3, SYT7) and 13 postsynaptic (CAMK2B, CAMKV, CPNE6, GABBR2, mGLUR2, HOMER1, OLFR1410, SGIP1, SHANK1, SSTR2, SYT3, TANC2, UNC80) factors (see Table S1).

Unbiased automated bioinformatics were employed to identify significant enrichments of protein–protein interactions and established pathways among the dysregulated factors. Indeed, the search tool for the retrieval of interacting genes (STRING) webtool confirmed bioinformatics enrichments: Among the downregulations (see Table S2A) significance was obtained for “Protein kinase C conserved region 2 (CalB)” among SMART protein domains (false discovery rate FDR = 4.09 × 10^−7^), for the “C2 domain” among INTERPRO and PFAM protein domains (FDR = 5.18 × 10^−7^) (illustrated as red bullets in Figure 3B), for the “Serine/threonine-protein kinase, active site” among INTERPRO domains (FDR = 9.14 × 10^−7^) (blue bullets), for the “Transmission across chemical synapses” (FDR = 8.78 × 10^−6^) (green bullets) with particularly “Glutamate binding, activation of AMPA receptors and synapses” (FDR = 1.74 × 10^−5^), and for the article “Bodaleo-FJ et al. 2016 Sci Rep; PMID: 27425640” on MAP1B-deficiency effects on synaptic components (purple bullets) [71]. Indeed, a total of 11 kinase domain factors showed dysregulation. Among the upregulations (Table S2B), significant enrichments were detected only for the expected terms “phosphoprotein” (FDR = 9.17 × 10^−14^) (yellow bullets in Figure 3C) and “neuron part” (FDR = 2.48 × 10^−5^) (light blue bullets).

Overall, the data identify novel phosphorylation effects of PINK1/SNCA within the microtubule support for the neuron-specific signaling machinery. 

### 2.4. Dysregulated Phosphorylation of pT1928-MAP1B and of Autophagy Factors in DM Mouse Brain

Beyond automated bioinformatics, it was noted that several autophagy factors showed differential phosphorylation and that these effects may correspond to the role of MAP1B as a positive cofactor in DAPK1-mediated autophagic vesicle formation and membrane blebbing [74,75]. MAP1B exists in a protein complex that may comprise its heavy-chain, its light-chain LC1, and also MAP1-LC3, which is encoded independently [76]. The LC3 subunit is essential for the formation of autophagosomes and the degradation of mitochondria in distal neuronal axons by the PINK1/Parkin pathway [77,78], while the MAP1B-LC1 complex can prevent autophagosome formation [79]. The MAP1B-LC1 complex was shown to assist the elimination of cell surface neuronal N-type Ca^2+^ (Ca_v_2.2) channels [80], and also Parkin influences the degradation of the Ca_v_2.2 channels [81], so it is interesting to note that pS783-CACNA1B showed a 2-fold increase in DM brain (Table S1). Furthermore, the MAP1B light-chain LC1 restricts the glutamate receptor AMPA membrane surface presence [82], so again, it is noteworthy that pS871 of the glutamate receptor mGluR2 showed a 2-fold increase (Table S1). Thus, all three subunits of the MAP1B-LC1-LC3 complex have a differential influence on the turnover of membrane receptors and mitochondria.

Consistent with this impact of MAP1B on DAPK1-dependent autophagy, a relevant phosphorylation deficit (−1.9-fold) occurred for DAPK2^S299^ in DM mouse brain (Table S1). Importantly, the VPS13 (Vacuolar Protein Sorting 13) family that regulates autophagy and is implicated in Parkinson’s disease or other neurodegenerative processes was also consistently altered. VPS13D^S2429^ showed a strong −3.0-fold downregulation, and VPS13C^S2480^ showed a −2.1-fold decrease. Mutations in VPS13D lead to neurodegeneration in the form of ataxia with spasticity, and VPS13D is required for the regulation of mitochondrial size, fission, and autophagic clearance [83,84]. Loss of VPS13C function was reported to cause early-onset autosomal recessive Parkinsonism and to increase PINK1/Parkin-dependent mitophagy [85]. Both show sequence homology to the yeast autophagy regulator ATG2 that mediates lipid transport [86]. 

Thus, it is unclear if the interaction between cytoskeletal components and the signaling machinery in the plasma membrane is crucial for PD pathogenesis, but clearly PINK1, Parkin, and VPS13D all function in the autophagic degradation of mitochondria and all were implicated as risk factors in the pathogenesis of PD. The above data support the notion that VPS13D phosphorylation is downstream from PINK1 kinase activity and A53T-SNCA neurotoxicity. 

Jointly, the data identify novel phosphorylation effects of PINK1/SNCA within the microtubule support for the autophagy machinery. 

### 2.5. Known PINK1/SNCA Functional Effects Mirror the Roles of Dephosphorylated Factors in DM Brain

The above phosphorylome profile from a PD mouse model cannot be easily validated by independent methods in other brains. PD patient autopsy tissue would show phosphorylation profiles with distortion due to postmortem delays and due to the loss of specific neuron projections. Frozen brain samples from patients with PINK1 mutation are not available. In the current absence of site-specific antibodies, the detection of the brain phosphorylation events documented above depends on peptide mass spectrometry again in quantitative label-free manner. In aged tissue homogenates, the cell surface accumulation of specific channels/pumps cannot be tested with precision. Furthermore, monitoring the time course or efficiency of mitochondrial autophagy in brain presents formidable challenges. Last but not least, the sizes of MAP1B protein (270 kDa) and ANK2 protein (426 kDa) make their comprehensive assessment in immunoblots and tryptic peptides a cumbersome task. 

Even so, it was already demonstrated that neuronal signaling and neurite extension are modified by PINK1 and alpha-synuclein mutations. In a neuronal cell model, the mitochondrial structure and neurite outgrowth were altered by co-expression of alpha-synuclein as stressor together with mutant PINK1 as abnormal stress-response [87]. The overexpression of human alpha-synuclein by itself resulted in reduced neurite extension [88]. This effect was mediated by physical interactions between alpha-synuclein and the cytoskeletal spectrin meshwork [89], and was dependent on altered activity of glycogen-synthase-kinase-3beta and protein phosphatase 2A [90]. Alpha-synuclein administration to striatal slices leads to a selective redistribution and activation of Ca_v_2.2 channels [91]. 

Primary mouse neurite retraction is also triggered by mitochondrial damage and PINK1 responses [92]. Dendrite outgrowth in primary neurons could be promoted by the overexpression of wildtype, but not mutant PINK1, even in the absence of its mitochondrial targeting sequence, via enhanced axonal transport of mitochondria and by a Protein-Kinase-A mediated mechanism [93]. In *Caenorhabditis elegans*, the mutation of PINK1 alone was sufficient to alter the mitochondrial structure and axonal outgrowth, phenotypes that could be rescued completely by the deletion of the LRRK2 ortholog [94]. Recently, it was shown that the levels of LRRK2 depend on PINK1 [95]. Thus, it is also conceivable as alternative possibilities that the PINK1 deletion is responsible directly for the deficient MAP1B and ANK2 phosphorylation, or indirectly via downstream LRRK2 kinase activity changes. It is noteworthy that LRRK2 contains an Ankyrin-repeat domain just like ANK2 and like DAPK1. Overall, it is unclear at present, which upstream mechanisms are responsible for the deficient phosphorylation of T1928-MAP1B and S3781-ANK2 in DM brains, while the downstream functional effects of SNCA/PINK1 are a good match with the roles of the dephosphorylated protein complexes. 

Overall, our novel molecular findings are in excellent agreement with previously known functional effects and phenotypes of PINK1/SNCA mutations.

### 2.6. Validation Tests Confirm PINK1 to Modulate MAP1B/ANK2, Indicate Decreased Autophagy in DM Brain

**Figure 4:**
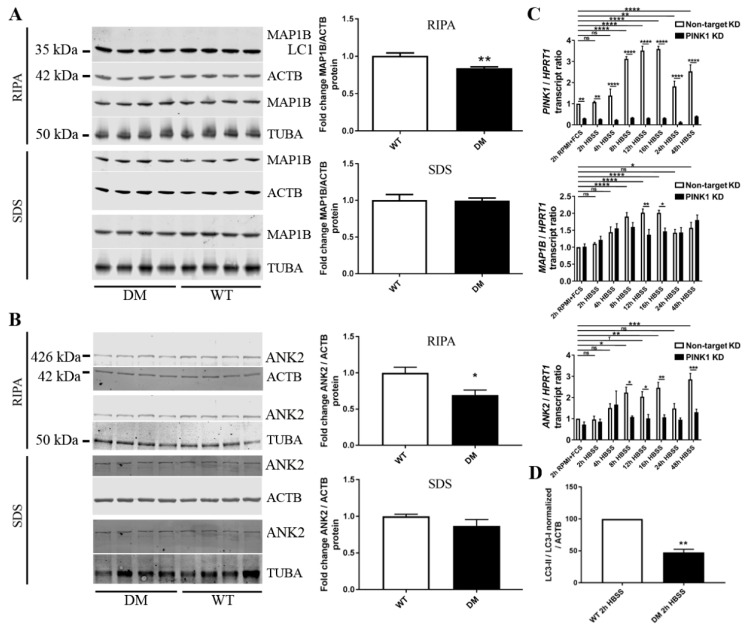
Validation experiments regarding expression modulation at protein and mRNA level. (**A**) Quantitative immunoblots detecting MAP1B relative to beta-actin (ATCB) as loading control and marker of the cortical actin cytoskeleton, or to alpha-tubulin (TUBA) as loading control and marker of the microtubule cytoskeleton, both for RIPA-soluble and for SDS-soluble fractions of the brain proteome. (**B**) Quantitative immunoblots detecting ANK2 relative to beta-actin (ATCB) as loading control or to alpha-tubulin (TUBA) as loading control, again for RIPA- and SDS-soluble fractions. (**C**) Transcript abundance of *PINK1* (above) and *MAP1B* (below), normalized to the loading control *HPRT1*, in human SH-SY5Y neuroblastoma cells undergoing nutrient deprivation in HBSS medium without fetal calf serum over 48 h. Black bars represent stable knock-down (KD) of *PINK1*, while white bars represent non-target (NT) knock-down control. Starvation induces *PINK1* mRNA in phasic manner with a peak at 12–16 h. *MAP1B* mRNA is induced in a similar pattern, but not sustained after 12 h in PINK1-KD cells (*n* = 4 *PINK1*-KD versus 4 NT-KD). (**D**) Quantitative immunoblots in primary cortical neurons (28 days in vitro) starved for 2 h with HBSS medium show a significant reduction of the LC3-II isoform versus LC3-I in DM mice (*n* = 4 DM versus 4 WT, two litters). Significance is illustrated by asterisks, with * *p* < 0.05, ** *p* < 0.01, *** *p* < 0.001, **** *p* < 0.0001.

To validate whether the massive phosphorylation deficits of MAP1B or ANK2 are indeed selective and are not based on a gross loss of protein abundance, quantitative immunoblots were performed in DM brain homogenate. MAP1B and ANK2 levels showed only modest reductions in the RIPA-soluble fraction that mostly contains cytosolic proteins, and no relevant change in the SDS-soluble fraction that contains mostly membrane proteins (Figure 4A,B). These reductions observed upon normalization versus beta-actin (a component of the cortical cytoskeleton) were even stronger upon normalization versus alpha-tubulin (a component of the microtubule cytoskeleton).

To obtain additional evidence whether PINK1 regulates MAP1B and ANK2, we chose to expose a human neural line to starvation stress (rather than to A53T-SNCA overexpression stress) and then examine the PINK1-dependent regulation of *MAP1B* and *ANK2* mRNA during a time-course experiment. A switch from RPMI + FCS medium to HBSS-FCS medium deprives cells of amino acids, serum lipids, and growth factors, providing minimal glucose levels, and potently leading to a several-fold increase in PINK1/Parkin expression [24]. As seen in Figure 4C, this nutrient restriction in SH-SY5Y neuroblastoma cells prompts a phasic two-fold induction of *MAP1B* mRNA with a maximum at 12 h latency, coinciding with the previously reported phasic induction of *PINK1* mRNA. However, this *MAP1B* transcriptional induction cannot be sustained after 8 h in cells with stable knockdown of *PINK1*, leading to significant deficits at 12 and 16 h. MAP1B has little expression in adult brain and is needed mostly when neural circuits develop and during regeneration periods [49,96], so our findings would suggest that MAP1B is less available and pathologically phosphorylated during stress response phases in PD models with PINK1 deficiency. Similarly, the expression of *ANK2* mRNA was induced two/three-fold by starvation stress with significance at 8 h, but remained elevated until 48 h. In cells with stable knockdown of *PINK1*, the starvation protocol failed to trigger *ANK2* mRNA induction, leading to significant genotype-dependent differences from 8 to 48 h. This massive dependence of *ANK2* induction on *PINK1* levels during starvation stress is very interesting in the light of two previous reports. Firstly, the deficiency of an Ankyrin-repeat containing *Drosophila* protein was shown to rescue mitochondrial pathology and phenotypes triggered by PINK1 and Parkin mutations [97]. Secondly, peptides derived from ANK2 were observed to be potent and specific inhibitors of autophagy via ATG8-binding [98]. 

It seems clear that neurite extension and neural signaling are altered in DM brains, but it remained questionable to what degree autophagy is abnormal. While several autophagy modulators, particularly VPS13C as PD risk factor, showed altered phosphorylation in DM brain, it cannot be distinguished whether this phosphorylome profile reflects pathology in the autophagic pathway or a successful homeostatic compensation. On the one hand, the A53T-SNCA overexpression would be predicted to enhance the autophagic degradation of protein aggregates, while on the other hand the formation of autophagosomes and the initiation of degradation might be impaired by PINK1 deletion via Beclin as well as by MAP1B/ANK2 dysfunction via LC3/ATG8. Thus, we used DM brain tissue to assess the conversion of LC3 from the microtubule-associated isoform I to the autophagosome-associated isoform II. Although the LC3-II/I ratio cannot predict how efficiently autophagosome will fuse with lysosomes and degrade the toxic material (therefore cell culture experiments with drugs like Bafilomycin A1 are recommended to test autophagic flux), in tissue and untreated cells, this ratio is a reliable marker of autophagosome abundance [99]. Again, HBSS medium was used to deprive DM primary cortical neurons from nutrients and thus trigger autophagy. After 2 h of starvation, quantitative immunoblots of DM cells showed a significantly smaller LC3II/I ratio (reduction to 48%, *p* = 0.0016) (Figure 4C). These findings suggest that the PINK1 deletion impairs autophagosome formation and the clearance of damaged mitochondria as well as of toxic protein aggregates. Our observations are in agreement with previous reports from a neuroblastoma cell line where PINK1 deficiency was impairing bulk autophagy [16]. The data also agree with previous findings that phosphorylated MAP1B is associated with autophagosomes [100] and that a reduction of MAP1B will attenuate DAPK1-mediated autophagy [74]. While LC3 is a requirement for autophagosome-mediated degradation, the LC1 fragment of MAP1B prevents autophagosome formation according to recent insights [79]. It would, therefore, be interesting in the future to generate neural cells with MAP1B-KnockIn of the phospho-dead T1928A residue versus the phospho-mimetic T1928E residue, to analyze whether absence of this specific phosphorylation event modulates the LC1 cleavage or the autophagosome association of MAP1B. 

Overall, the validation experiments confirm a modulatory influence of PINK1 on MAP1B as well as on ANK2. In addition, the autophagic impairment observed in the DM neurons is also explained as a putative consequence of the PINK1 deletion.

## 3. Materials and Methods

### 3.1. Breeding and Ageing of DM Mice, Homozygous for *Pink1^−^*^/−^ and A53T-SNCA Overexpression

We previously described the generation, ageing, and characterization of DM mice [46]. They contain 129/SvEv and FVB/N genetic backgrounds approximately in a 50:50 distribution. As WT control mice, we used aged F1-hybrids from a crossbreeding of 129/SvEv and FVB/N mice, which were descended from littermates of the respective single mutant animals. Pairs of DM and WT mice with matched age and male sex were kept in individually ventilated cages under 12 h light cycle with food and water ad libitum. Sentinel mice with regular health monitoring including blood tests for viral and parasite infections detected no pathology. The mice under investigation were bred and aged at the FELASA-certified Central Animal Facility (ZFE) of the Frankfurt University Medical School. Housing of animals was in accordance with the German Animal Welfare Act, the Council Directive of 24 November 1986 (86/609/EWG) with Annex II, and the ETS123 (European Convention for the Protection of Vertebrate Animals). Our institutional review board, the Regierungspraesidium Darmstadt, approved our project V54-19c20/15-FK/1083 on 27 March 2017.

### 3.2. Global Phospho-Ser/Thr Motif Survey by Label-Free Mass-Spectrometry

After decapitation, brain hemispheres from 18-month-old mice (three DM versus three WT animals, matched for male sex) were removed in parallel, snap frozen in liquid nitrogen, stored at −80 °C, and transported on dry ice to Cell Signaling Technology, Inc. (Danvers, MA 01923, USA) for an outsourced PhosphoScan^®^ procedure [101,102]. In brief, brain extracts were trypsin-digested and subjected to C18 solid-phase extraction. The lyophilized peptides were immunoprecipitated by protein-A/G-agarose-immobilized mix of Phospho-Ser/Thr antibodies (Cell Signaling Technology, Inc., Danvers, MA 01923, USA). The procedures were performed as in the ubiquitylome study on such brains [46]. This procedure yielded a total of 7508 redundant phosphorylated peptide assignments to 3526 unique phosphorylated peptides. The quantitative data from the three control WT mice were averaged to compare each DM mouse individually and derive the respective fold-change. There was no normalization to a housekeeping phospho-site, but median offset correction normalization with log_2_(ratio) values. The original data including filtering criteria regarding retention time, Xvorr value, mass accuracy, DeltaCN, Rsp value, PP probability, charge states, intensity, area, %CV, and MS/MS quality are shown in Suppl. Table S1 or are available from the authors upon request.

### 3.3. Bioinformatic Pathway Enrichment Analyses

For protein–protein-interaction (PPI) network analysis, the software tool STRING (search tool for the retrieval of interacting genes) v.11.0 (EMBL, Heidelberg, Germany, https://string-db.org/) with standard settings was used to visualize networks among factors with >2-fold dysregulation in at least one of the three biological replicates [103]. Automated network statistics were performed; significant functional enrichments of GO (gene ontology terms regarding biological processes, molecular functions, cellular components), KEGG pathways, Reactome pathways, PFAM protein domains, INTERPRO Protein Domains and Features, and SMART protein domains were exported into Excel files.

### 3.4. Validation Experiments via Expression Analysis on Protein and mRNA Level

Quantitative immunoblots of MAP1B were performed as in the previous description of the DM mice [46], employing the anti-MAP1B antibody from BD Biosciences (San Jose, CA, USA, catalog # 612678), the anti-ACTB antibody from Sigma (Ronkonkoma, NY, USA, # A5441), and the anti-TUBA antibody from Abcam (Cambridge, UK, # ab15246) at the recommended dilutions. Quantitative RT-PCR was carried out with Taqman assays from Thermofisher (Waltham, MA, USA, for *PINK1* Hs00260868-m1, for *MAP1B* Hs00195485-m1, for *HPRT1* Hs99999909-m1) again as described in [46]. Quantitative immunoblots of LC3 isoforms with the anti-LC3 antibody from Sigma (Ronkonkoma, NY, USA, # L8918) and the culture of primary cortical neurons from 1–4-day-old mice with Neurobasal medium over 4 weeks were also done as reported [16].

## 4. Conclusions

DM mouse brains with A53T-SNCA overexpression as stressor and with PINK1 deletion to impair stress-responses were employed in a pioneer Ser/Thr-phosphorylome survey by quantitative label-free mass spectrometry. This approach documented a prominent massive deficit of pT1928-MAP1B, a strong deficit of pS3781-ANK2, and a cluster of dephosphorylation events at cytoskeletal anchors of the synaptic signaling machinery, of mitochondria, or of autophagosomes. The MAP1B and ANK2 dephosphorylation sites show strong phylogenetic conservation and adhere to the sequence preference of PINK1 kinase. Validation experiments provided additional evidence that MAP1B is modulated by PINK1 and that reduced autophagosome availability exists in DM neurons. Our findings, for the first time, identify neuron-specific factors that are regulated in similar manner as other known ubiquitous mitophagy and cytoskeleton components, thus shedding light on the selective vulnerability of aged neuron populations. Preferential expression in neurons is particularly known for ANK2, 4.1N, and ADD2, but is somewhat true also for MAP2, MARK1, MAP1A, and KIF1A. In contrast, mitochondrial components like VDAC3, or cytosolic factors like MIRO1, MAP1B, VPS13C, and CLASP1 are similarly expressed in many different tissues. The dephosphorylated factors in DM brain have roles, which are in good agreement with previously observed functional deficits of PD models with PINK1 deficiency or/and SNCA neurotoxicity. Overall, the documented Ser-/Thr-phospho-profile may be useful as biomarker to assess the benefit of novel neuroprotective drugs, but also to identify crucial molecular targets of preventive treatment. It is important to note that MAP1B is already known as physical interactor protein of alpha-synuclein, is also known as target of LRRK2 phosphorylation, and now appears a candidate for PINK1-dependent phosphorylation. Furthermore, ANK2 genetic variants contribute to the risk to develop PD and given that ANK2 also appears as potential target of PINK1-dependent phosphorylation, we now propose to conduct further investigations into the role of MAP1B with its T1828 residue and ANK2 with its S528 residue in the pathogenesis of PD.

## Figures and Tables

**Table 1:** Phosphoproteomics revealed a cluster of dysregulations (downregulations in blue, upregulations in red color) in factors associated with microtubules and microfilaments. The list also presents signal intensity and coefficient of variance (CV), the phosphorylation site, the peptide sequence around the phosphorylated residue (illustrated by asterisk *), and the peptide count. A selective massive deficit of pT1928 in microtubule-associated protein 1B (MAP1B) and the second strongest deficit of pS3781 in ANK2 as a MAP1B-interactor were documented. These prominent changes clustered with an enrichment of factors that link the cytoskeleton to either the mitochondria or to the plasma membrane signaling apparatus, namely MAP2, EPB41L2 (4.1G), MARK1, MAP1A, KIF1A, RP1, ADD2, CLASP1, and EPB41L1 (4.1N), see Figure 3A.

Normalized Fold Change					Max % CV					
DM all: Control	DM 1: Control	DM 2: Control	DM 3: Control	Max Intensity	Biological	Gene Name	Protein Name	Site	Peptide	Count in Details
−314.3	−255.3	−438.7	−279.5	6,559,198	35.4	Mtap1b	MAP1B	§1928	TIKT*PEDGGYTCEITEK	16
−3.3	−543.3	−715.4	−1.1	858,179	155.1	Ank2	ANK2 iso2 + iso3	§3781; §3809; §872	GSPIVQEPEEASEPKEESS*PRK	29
−3.0	-1.0	−47.8	−31.0	947,741	143.6	Vps13d	VPS13D	§2429	NAS*SESAVVPK	8
−2.3	−2.9	−2.9	−1.6	26,223	54.5	Mtap2	MAP2 iso6	§1801; §471	RLSNVSSS*GSINLLESPQLATLAEDVTAALAK	3
−2.3	−2.9	−2.9	−1.6	26,223	54.5	Mtap2	MAP2 iso6	§1803; §473	RLSNVSSSGS*INLLESPQLATLAEDVTAALAK	3
−2.1	−2.1	−3.1	−1.4	436,205	32.6	Epb4.1l2	EPB41L2 iso3	§86	QRS*YNLVVAK	3
−1.9	−4.5	−1.5	−1.4	543,218	47.1	Dapk2	DAPK2	§299	RES*VVNLENFKK	11
−1.8	−2.2	−1.4	−2.0	27,656	33.9	Mark1	MARK1	§394, §403	SRPSS*DLNNSTLQS*PAHLK	19
−1.7	−2.1	−1.7	−1.4	405,747	33.8	Vps13c	VPS13C	2481	QESS*LFTLTFVPYGYTEVASVPVAR	5
−1.6	−1.2	−5.2	−1.1	400,265	53.3	Mtap1a	MAP1A	§1794	VPSAPGQESPVPDT*KSTPPTR	2
−1.6	−1.2	−5.2	−1.1	400,265	53.3	Mtap1a	MAP1A	1796	VPSAPGQESPVPDTKS*TPPTR	2
−1.6	−1.2	−5.2	−1.1	400,265	53.3	Mtap1a	MAP1A	§1797	VPSAPGQESPVPDTKST*PPTR	31
−1.6	−2.1	−1.4	−1.4	2,098,225	24.4	Kif1a; Kif1a	KIF1A iso3	§1537; §1540	SRPAS*PEPELLPELDSK	22
−1.4	−1.1	−2.6	-1.1	454,218	35.3	Mtap1a	MAP1A	§1789, 1796	VPSAPGQES*PVPDTKS*TPPTR	3
−1.4	−1.1	−2.6	−1.1	454,218	35.3	Mtap1a	MAP1A	§1789, §1797	VPSAPGQES*PVPDTKST*PPTR	43
1.5	2.0	1.5	1.1	674,946	30.3	Mapre2	RP1	§218, §222	SSPASKPGSTPS*RPSS*AK	11
1.6	2.3	1.5	1.1	563,552	32.4	Mapre2	RP1	§215, §222	SSPASKPGS*TPSRPSS*AK	2
1.7	1.8	2.1	1.3	12,258,948	25.6	Add2	ADD2	§528, §532, §535	S*RS*PS*TES*QLMSK	26
1.7	2.2	1.9	1.2	519,692	28.2	Mark1	MARK1	§504	RNT*YVCER	8
1.7	2.0	1.9	1.2	39,365	30.1	Clasp1	CLASP1 iso3	§646, §649	RQS*SGS*TTNVASTPSDSR	5
2.2	1.2	4.4	1.0	1,375,910	82.0	Epb4.1l1	EPB41L1 iso2 + iso4	§546; §545	RLPSSPASPS*PK	29

## Supplementary Materials

Supplementary materials can be found at https://www.mdpi.com/1422-0067/20/13/3284/s1. Supplementary Table S1: Global mouse brain proteome phosphorylation with >2-fold dysregulation at least once among the biological triplicates, illustrating downregulations in red and upregulations in green, and emphasizing proteins with consistent >2-fold changes by a more intense coloring. Relevant bioinformatics and annotations for each phosphorylation site are provided in separate datasheets. Supplementary Table S2: STRING Heidelberg database pathway enrichments among the proteins (A) of all downregulated and (B) of all upregulated phospho-sites.
